# Postoperative respiratory depression after hysterectomy

**DOI:** 10.17305/bjbms.2020.5026

**Published:** 2021-06

**Authors:** Mariana L. Laporta, Michelle O. Kinney, Darrell R. Schroeder, Juraj Sprung, Toby N. Weingarten

**Affiliations:** 1 Department of Anesthesiology and Perioperative Medicine, Mayo Clinic, Rochester, Minnesota, USA,; 2 Department of Health Sciences Research, Division of Biomedical Statistics and Informatics, Mayo Clinic College of Medicine and Science, Rochester, Minnesota, USA

**Keywords:** Postoperative respiratory depression, postoperative complication, anesthesia recovery period, opioid-induced respiratory depression, hysterectomy

## Abstract

To investigate if sex-specific physiologic characteristics could impact postoperative respiratory depression risks in women, we studied incidence and risk factors associated with postoperative respiratory depression in a gynecologic surgical cohort. Only hysterectomies performed under general anesthesia from 2012 to 2017 were included to minimize interprocedural variability. Respiratory depression was defined as episodes of apnea, hypopnea, hypoxemia, pain-sedation mismatch, unplanned positive airway pressure device application, or naloxone administration in the post-anesthesia care unit. Multivariable logistic regression was used to explore the association with clinical characteristics. From 1974 hysterectomies, 253 had postoperative respiratory depression, yielding an incidence of 128 (95% confidence interval [CI], 114–144) per 1000 surgeries. Risk factors associated with respiratory depression were older age (odds ratio 1.22 [95% CI 1.02–1.46] per decade increase, *p*=0.03), lower body weight (0.77 [0.62–0.94] per 10 kg/m^2^, *p* = 0.01), and higher intraoperative opioid dose (1.05 [1.01–1.09] per 10 mg oral morphine equivalents, *p* = 0.01), while sugammadex use was associated with a reduced risk (0.48 [0.30–0.75], *p* = 0.002). Respiratory depression was not associated with increased hospital stay, postoperative complications, or mortality. Postoperative respiratory depression risk in women increased with age, lower weight, and higher intraoperative opioids and decreased with sugammadex use; however, it was not associated with postoperative pulmonary complications.

## INTRODUCTION

Postoperative respiratory depression is a pulmonary complication resulting from both decreased respiratory drive and upper airway obstruction and is typically secondary to sedating medications [1]. Respiratory depression during immediate anesthesia recovery in the postanesthesia care unit (PACU) is common [2], and while oftentimes believed to be self-limited, has been associated with serious postoperative pulmonary complications which can lead to severe morbidity and mortality [2,3]. Previous investigations on postoperative respiratory depression have found increased risk with male sex, obstructive sleep apnea, and gabapentinoids [2,4,5]. However, compared to men, women have a lower prevalence of obstructive sleep apnea [6,7] and typically lower postoperative analgesic requirements [8]; two characteristics which may be protective against post-operative respiratory depression. Gabapentin and pregabalin are frequently used as a part of multimodal pain therapy in gynecological procedures [9]. However, gabapentinoids have recently been recognized to lead to respiratory depression when used with other sedating medications [5]. Therefore, the incidence and risk factors for postoperative respiratory depression might exhibit sex-specific characteristics. Although postoperative pulmonary complications following hysterectomy are infrequent (<4%) [10,11], given that over 600,000 women undergo this procedure annually in the United States [12], a substantial number may still be affected. The primary aim of this study was to determine the incidence and risk factors for postoperative respiratory depression among women undergoing hysterectomy during anesthesia recovery in the PACU. A secondary aim was to compare postoperative outcomes among patients who did or did not have respiratory depression during anesthesia recovery.

## MATERIALS AND METHODS

This study was approved by the Mayo Clinic Institutional Review Board (protocol No. 19-002835, approved on July 25, 2019). Consistent with Minnesota Statute 144.295, at enrollment, all participants had provided prior written authorization for medical records use in retrospective studies.

### Study setting

This study was done in a major academic facility.

### Study design

This retrospective observational study was designed to determine the incidence, risk factors, and outcomes of respiratory depression during anesthesia recovery following hysterectomy with general anesthesia.

### Patient selection

Adult women who provided prior written research authorization, underwent primary hysterectomy under general anesthesia, and were admitted to the PACU from January 01, 2012, to December 31, 2017, were included in the study. Patients undergoing emergent procedures, combined obstetric procedures, and extensive combined procedures (e.g., pelvic exenteration, liver resection, nephrectomy, bowel resection, and omentectomy) were excluded.

### Perioperative practice

All cases were performed under general anesthesia. Analgesia management was multimodal, consisting of opioid, acetaminophen, non-steroid anti-inflammatory drugs (celecoxib 200 mg orally before surgery or 15 mg ketorolac intravenous at end of surgery), ketamine, and gabapentin. However, this management was not part of a protocol and left to the discretion of individual anesthesiologists. All patients were administered neuromuscular blocking drugs to facilitate surgical exposure, typically vecuronium or rocuronium, which was reversed at the conclusion of surgery with either neostigmine combined with glycopyrrolate or sugammadex. At the end of the surgery, tracheas were extubated in the operating room, and all patients were discharged to the PACU.

The PACU is staffed by registered nurses trained in phase I anesthesia recovery with the attending anesthesiologist readily available when advanced expertise is required. The nurses continuously monitor the patient for pain scores (based on a standard 11-point verbal pain scale [0 = no pain, 10 worst pain imaginable]), sedation scores (Richmond Agitation Sedation Scale [RASS] [13]), and postoperative signs of respiratory depression or “respiratory specific events” (hypoventilation [three episodes of <8 respirations/min]; apnea [episode of apnea of ≥10 s]; hypoxemia [3 episodes of capillary oxygen saturation <90% or <preoperative saturation with or without oxygen]; episodes of moderate pain despite high sedation, or “pain-sedation mismatch” [verbal pain scale >5 with RASS≤3]) [2]. Patients who are witnessed to have a respiratory specific event have their phase I recovery prolonged by at least 60 minutes for increased monitoring and may require intervention with naloxone or application of positive airway pressure devices. Also, standard PACU discharge criteria are used [14], and time achieved is precisely documented and used to determine anesthesia recovery duration.

### Data collection

Electronic medical records were abstracted using automated data software as previously described [15], and manually confirmed. Baseline characteristics included demographics, the burden of comorbid conditions (determined by the Charlson comorbidity score [16]), and obstructive sleep apnea assessment [2]. Perioperative variables included surgical approach and duration, anesthetic medications, PACU course, postoperative complications, length of hospital stay, rapid response team activation, and mortality related to the procedure. Respiratory depression in the PACU was defined as the occurrence of respiratory specific events, as well as any unplanned use of positive airway pressure devices, naloxone administration, or reintubation for respiratory failure. Oral morphine equivalents (OMEq) were calculated using standard conversions [17]. Severe pain was defined as a verbal pain score of ≥7, over-sedation was defined as a RASS score ≤−3, and postoperative nausea and vomiting (PONV) was defined if a rescue antiemetic was administered.

### Data analysis

Data are presented as median (interquartile range) or mean±standard deviation for continuous variables and as the number of patients (percentage) for categorical variables. The primary endpoint was a binary variable indicating the occurrence of respiratory depression in the PACU. We compared patients with and without respiratory depression using the Student’s t-test or rank-sum test for continuous variables and the Chi-square test for categorical variables. In addition, multivariable logistic regression analysis was performed to assess potential associations between patient and procedural characteristics and respiratory depression in the PACU (Table 1). Potential confounders included in the analysis were based on existing literature and previous studies from our institution [4,18-22]. Comparisons of outcomes between patients who had respiratory depression and those who did not were made using descriptive statistics. A two-tailed *p*<0.05 was considered statistically significant. Statistical analyses were performed with statistical software (JMP Pro version 13.0.0, Cary, NC, USA).

**TABLE 1:**
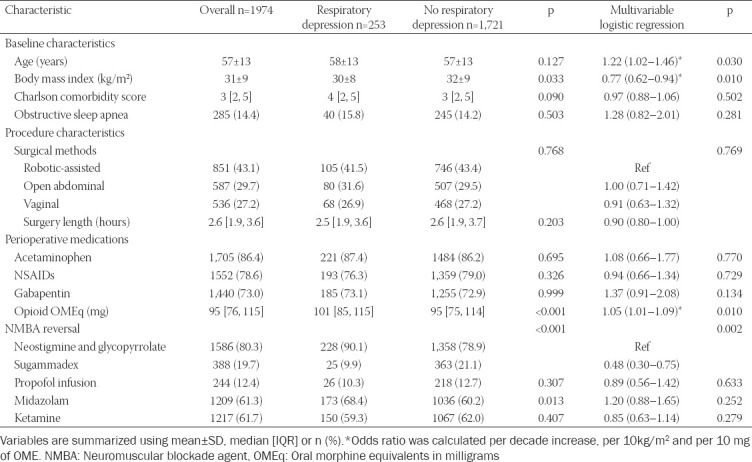
Patient and procedure characteristics

## RESULTS

During the study time-frame, 1974 hysterectomies were performed under general anesthesia, with 803 (62.9%) total hysterectomies combined with bilateral salpingo-oophorectomy. The surgical approach was robotic-assisted in 851 (43.1%) patients , laparotomy in 587 (29.7%) patients, and vaginal in 536 (27.2%) patients. Two hundred fifty-three patients had respiratory depression in the PACU, yielding an incidence of 128 (95% confidence interval [CI] 114–144) per 1000 surgeries, and consisted of 162 (8.2%) episodes of hypoventilation, 94 (4.8%) episodes of hypoxemia, 75 (3.8%) episodes of apnea, 8 (0.4%) cases of pain-sedation mismatch, 2 (0.1%) cases of unplanned application of positive airway pressure devices, and 11 (4.3%, incidence 5.6 [3.1–10.0] per 1000 cases) naloxone administrations (median [25%, 75% quartile] dose 0.4 [0.4–0.8] mg). No patient required reintubation in the PACU.

Clinical variables between patients who had or did not have PACU respiratory depression are presented in Table 1. Multivariable analysis was performed with the variables listed in Table 1, and respiratory depression was found to be associated with older age (odds ratio 1.22, 95% CI 1.02–1.46 per decade of life, *p*=0.03), lower body weight (0.77, 95% CI 0.62–0.94 per 10 kg/m^2^ of body mass index, *p*=0.01), and higher intraoperative opioid dose (1.05, 95% CI 1.01–1.09 per 10 mg OME, *p*=0.01). Reversal of muscle relaxant with sugammadex, as opposed to neostigmine with glycopyrrolate, was also associated with a lower risk for respiratory depression (0.48, 95% CI 0.32–0.82, *p*=0.002).

Postoperative outcomes between the groups are summarized in Table 2. Per practice protocol (see methods), patients with respiratory depression had longer PACU stay (median [IQR] 2.6 [2.0, 3.3] vs. 1.5 [1.1, 2.1] hours, *p*<0.001). These patients were also more sedated. Patients with respiratory depression also had higher rates of severe pain and PONV. Twenty-three patients developed postoperative pulmonary complications, which did not differ between patients who did or did not have respiratory depression in the PACU; however, these complications were more frequent following open abdominal procedures (13 [2.2%] vs. robotic-assisted 7 [0.8%] vs. vaginal 3 [0.6%], *p*=0.02). There were also 22 rapid response team activations, which did not differ between respiratory depression groups. Only one of these rapid response team activations was for opioid-induced respiratory depression, an 83-year-old woman who underwent a total abdominal hysterectomy. She had signs of respiratory depression in the PACU (apnea and hypopnea), resulting in an extended PACU stay for monitoring and supplemental oxygen. After an hour of normal respiratory function, the anesthesia team assessed she was safe for discharge to the ward. Four hours later, she became somnolent, hypopneic, and was noted to have pin-point pupils; a rapid response team was activated, and she was administered 0.4 mg of naloxone and fully regained normal breathing and alertness without any other complications during hospitalization. Only one in-hospital death occurred, a 68-year-old woman who underwent a total abdominal hysterectomy for metastatic carcinoid complicated by enterocutaneous fistula, peritonitis, sepsis, cardiogenic shock, and died on the postoperative day 62.

**TABLE 2:**
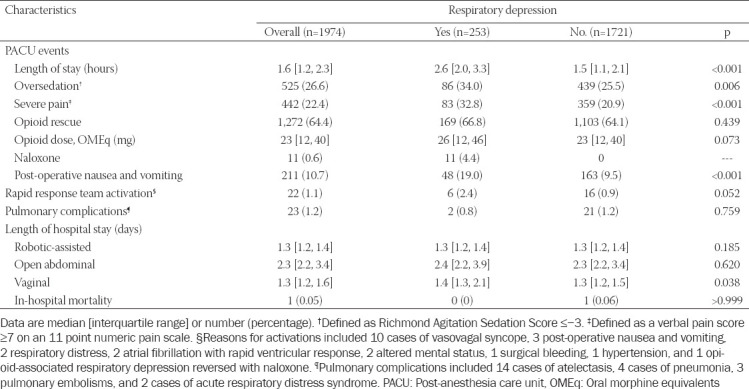
Perioperative outcomes

Based on safety concerns of gabapentin in multimodal anesthesia, we performed a *post hoc* analysis of its association with other PACU outcomes. Gabapentin was associated with increased rates of over-sedation (1.58, 95% CI 1.25–2.01, *p*<0.001), but not associated with a reduction in the rates of severe pain (0.81, 95% CI 0.64–1.02, *p*=0.089).

## DISCUSSION

The approximate rate of respiratory depression following hysterectomy with general anesthesia was 12%. The most important finding is that the risk for respiratory depression was reduced with use of neuromuscular blockade reversal with sugammadex and increased with older age, lower body weight, and higher use of opiates intraoperatively. These episodes of respiratory depression were not associated with increased risk for pulmonary complications during hospitalization.

Similar to our previous mixed sex-cohort studies [4,18,20], we found in this female surgical cohort that older age, lower body weight, and a higher opioid dose increased risk for postoperative respiratory depression. Older age and lower body weight could indicate frailty; however, the risk was not associated with the Charlson comorbidity index. Studies have suggested that among women, opiates have higher analgesic potency, slower action onset and offset, and more impact on ventilatory control [23,24]. Differing from our previous studies [4,18-20], OSA was not associated with increased risk, raising the possibility of sex-specific interaction between OSA and postoperative respiratory depression. Supportive of this theory was our earlier observation of low rates of postoperative respiratory depression (4.5%) in a bariatric surgical cohort, which consisted of 78% women with an OSA rate of 64% [25]. Also, contrasting from earlier studies [4,18,19,26], perioperative gabapentin was not associated with increased risk for respiratory depression; however, it was associated with increased rates of over-sedation in the PACU, which itself could increase the risk of respiratory complications in higher-risk patients [27].

Interestingly, sugammadex reversal of neuromuscular blockade was associated with a lower risk of post-operative respiratory depression. Recently, a large multicenter mixed-sex study found that sugammadex reversal, compared to neostigmine, reduced the risk of postoperative pulmonary complications by 30% and respiratory failure by 55% [28]. Two other recent studies found sugammadex decreased postoperative hypoxic episodes and intervention rates for postoperative respiratory failure, reducing both postoperative reintubation and non-invasive ventilation requirements [29,30]. The apparently emerging benefit of this relatively newer reversal agent will need to be examined in future prospective trials.

In this study, the rate of respiratory depression in PACU (12%) was substantially lower than our previous report of mixed-sex patient population undergoing lower extremity arthroplasty under general anesthesia (31%) [4], but similar to that observed after various laparoscopic operations (15%) [18], and higher than the rate observed following bariatric surgery [25]. While most episodes of respiratory depression in this study were self-limited, more serious episodes required interventions such as naloxone administration. The rate of naloxone administration in this study (0.6%) was similar to the rate (0.5%) observed in our study of respiratory depression following laparoscopic procedures [18]. It was unexpected that PACU respiratory depression rates would be similar among the three surgical approaches (robotic, vaginal, and laparotomy), though the laparotomy was associated with increased risk for postoperative pulmonary complications.

Per practice guidelines, patients in this study who had respiratory depression in PACU had longer anesthesia recovery. These patients also had higher rates of severe pain and PONV. Whether this longer PACU stay contributed to these other complications is not clear. Further, patients with severe pain may have been administered more opioids, leading to subsequent respiratory depression. Unfortunately, causality cannot be distinguished from our retrospective data. Fortunately, postoperative complications were not increased among patients with respiratory depression in the PACU, differing from our previous reports where these episodes were strongly correlated with serious episodes of opioid-induced respiratory depression on the wards [19,21].

### Limitations

This study has all limitations related to its retrospective design and potential errors associated with automated data extraction. The use of nurse-diagnosed respiratory specific events relies on witnessing and recognizing signs of respiratory depression and therefore is a somewhat subjective measure. Lastly, the anesthetic management was left to the discretion of the supervising anesthesiologist, introducing the possibility of treatment bias (e.g., using a more protective anesthetic with shorter-acting agents in patients deemed higher risk).

## CONCLUSION

In conclusion, respiratory depression in the PACU is associated with increased age, lower body mass index, and greater intraoperative opioid dose. The neuromuscular blockade reversal with sugammadex, as opposed to neostigmine, was associated with decreased risk for respiratory events in the recovery room.
